# Retraction Note: Silencing of Glutathione S-Transferase Pi Inhibits Cancer Cell Growth via Oxidative Stress Induced by Mitochondria Dysfunction

**DOI:** 10.1038/s41598-020-77205-9

**Published:** 2020-11-19

**Authors:** Naoki Fujitani, Akihiro Yoneda, Motoko Takahashi, Akira Takasawa, Tomoyuki Aoyama, Tadaaki Miyazaki

**Affiliations:** 1 Department of Biochemistry, Sapporo Medical University School of Medicine, S1W17, Sapporo, 060-8556 Japan; 2 Department of Molecular Therapeutics, Center for Food and Medical Innovation, Institute for the Promotion of Business-Regional Collaboration, Hokkaido University, N21W11, Sapporo, 001-0021 Japan; 3 Department of Pathology, Sapporo Medical University School of Medicine, S1W17, Sapporo, 060-8556 Japan; 4 Department of Surgical Pathology, Sapporo Medical University School of Medicine, S1W16, Sapporo, 060-8556 Japan; 5 Department of Probiotics Immunology, Institute for Genetic Medicine, Hokkaido University, N15W7, Sapporo, 001-0015 Japan

Retraction of: *Scientific Reports* 10.1038/s41598-019-51462-9, published online 14 October 2019

The Authors are retracting this Article. The Authors did not have permission for the use of the human cancer cell line M7609 included in this study.

All Authors agree with the retraction and its wording.

